# Phenotypic and Genetic Variability of Isolates of ZIKV-2016 in Brazil

**DOI:** 10.3390/microorganisms10050854

**Published:** 2022-04-21

**Authors:** Lidiane Menezes Souza Raphael, Iasmim Silva de Mello, Mariela Martínez Gómez, Ieda Pereira Ribeiro, Nathália Dias Furtado, Noemia Santana Lima, Alexandre Araújo Cunha Dos Santos, Déberli Ruiz Fernandes, Stephanie Oliveira Diaz da Cruz, Luana Santana Damasceno, Patrícia Brasil, Myrna Cristina Bonaldo

**Affiliations:** 1 Laboratory of Molecular Biology of Flavivirus, Oswaldo Cruz Institute, Fiocruz, Rio de Janeiro 21040-900, Brazil; lidiane.raphael@ioc.fiocruz.br (L.M.S.R.); iasmimmello@aluno.fiocruz.br (I.S.d.M.); ieda.ribeiro@ioc.fiocruz.br (I.P.R.); nathaliafurtado@aluno.fiocruz.br (N.D.F.); noemiasl@gmail.com (N.S.L.); xandeacs@ioc.fiocruz.br (A.A.C.D.S.); deberlifernandez@aluno.fiocruz.br (D.R.F.); stephanieodc@gmail.com (S.O.D.d.C.); 2 Molecular Biology and Genetics Division, Molecular Biology Department, Clemente Estable Biological Research Institute, Montevideo 11600, Uruguay; marielamartinezgomez@gmail.com; 3 Laboratory of Acute Febrile Diseases, National Institute of Infectious Diseases Evandro Chagas, Fiocruz, Rio de Janeiro 21040-900, Brazil; luanadamasceno383@gmail.com (L.S.D.); patricia.brasil@ini.fiocruz.br (P.B.)

**Keywords:** Zika virus, breast milk, virus isolation, non-structural proteins, biological characterization, virulence

## Abstract

The possibility of a Zika virus epidemic resurgence requires studies to understand its mechanisms of pathogenicity. Here, we describe the isolation of the Zika virus from breast milk (Rio-BM1) and compare its genetic and virological properties with two other isolates (Rio-U1 and Rio-S1) obtained during the same epidemic period. Complete genomic analysis of these three viral isolates showed that they carry characteristics of the American isolates and belong to the Asian genotype. Furthermore, we detected eight non-synonymous single nucleotide variants and multiple nucleotide polymorphisms that reflect phenotypic changes. The new isolate, Rio-BM1, showed the lowest replication rates in mammalian cells, induced lower cell death rates, was more susceptible to treatment with type I IFN, and was less pathogenic than Rio-U1 in a murine model. In conclusion, the present study shows evidence that the isolate Rio-BM1 is more attenuated than Rio-U1, probably due to the impact of genetic alterations in the modulation of virulence. The results obtained in our in vitro model were consistent with the pathogenicity observed in the animal model, indicating that this method can be used to assess the virulence level of other isolates or to predict the pathogenicity of reverse genetic constructs containing other polymorphisms.

## 1. Introduction

Zika virus (ZIKV) is an arbovirus that was discovered in 1947 in Uganda. After being silently endemic in Africa for many years, it spread through other parts of the world, causing outbreaks in the Yap Islands of the Federated States of Micronesia [1], French Polynesia [2], and Oceania. In late 2015, Brazil reported the first known local transmission of ZIKV in the Americas [3]. During its dissemination, the evolution of the virus resulted in increased pathogenicity [4,5]. Although it had been previously associated only with mild disease [1], after reaching the Americas the ZIKV infection was reported to cause neuropathology, including fetal brain development disorders and Guillain-Barré syndrome [5,6,7].

The ZIKV classical transmission occurs mainly through mosquito bites from *Aedes albopictus* and *Aedes aegypti* [8]. However, there are non-vector modes of transmission, such as mother-to-child transmission (MTCT) (congenital, perinatal, and postnatal), and sexual transmission [9,10,11]. RNA detection and ZIKV isolation from different biological fluids such as urine, saliva, and breast milk have been described [12,13]. In addition, RNA and infective ZIKV particles have been detected in breast milk samples from infected women and in serum from their respective children [12,14,15,16,17]. Four cases of probable MTCT of ZIKV through breast milk have been reported [10,12,18], suggesting that viral transmission during breastfeeding could have raised the number of infected infants during the ZIKV epidemic in Latin America (2015–2016) [19]. Even though the number of cases reporting the presence of ZIKV in this fluid is increasing, breastfeeding is recommended due to the benefits of this practice overcoming the potential harms. Furthermore, although Zika virus malformations occur in congenital infection, similar malformations have not been reported in children [20].

ZIKV has a positive-sense, single-stranded RNA genome of approximately 11 kilobases in length [21]. The genome contains 5′ and 3′ untranslated regions (UTRs) flanking a single open reading frame (ORF) that encodes a precursor polyprotein that is cleaved into three structural proteins: the capsid (C), pre-membrane/membrane (prM), and envelope (E), and seven non-structural proteins (NS1, NS2A, NS2B, NS3, NS4A, NS4B, and NS5) [21,22]. Phylogenetic analysis revealed two major ZIKV lineages: the African and the Asian [23]. Genome sequence analysis showed that the ZIKV strains currently circulating in South America share >99% identity with ZIKV isolates from French Polynesia outbreaks and belong to the Asian lineage [24]. Strains from these outbreaks seem to be associated with neurological damage, including a few reported cases of bilateral macular and peri-macular lesions [5], demonstrating that ZIKV genetic evolution has resulted in expanded viral tropism and diverse pathogenicity, but a recent study showed that African ZIKV strains could also cross the placenta and cause adverse perinatal outcomes, such as fetal loss rather than congenital disabilities [25].

A decrease in ZIKV transmission has been observed in the last three years, and human cases are sporadically detected, introducing the possibility of a new epidemic event [26]. A better understanding of the contribution of viral genetic variants to the infectious process is of paramount importance for designing prophylactic or therapeutic strategies to control ZIKV infections. Here, we describe the isolation of ZIKV from human breast milk (Rio-BM1), a biological fluid that deserves attention, and present a comparative genetic and biological characterization of three ZIKV isolates from the metropolitan region of Rio de Janeiro selected during the same epidemic period of 2016. Phylogenetic analysis demonstrated that the isolates reported in this manuscript belong to the Asian lineage, American strain. We identified non-synonymous mutations in the genomes of these ZIKV isolates that might be related to differences observed in virological properties, such as growth rates in mammalian cell lines, plaque size morphology, cell death induction, sensitivity to type-I interferon (IFN) treatment, and pathogenesis in a murine model. Finally, our results suggest a higher degree of attenuation of the ZIKV Rio-BM1 isolate as compared to the ZIKV isolated from urine, Rio-U1 isolate.

## 2. Materials and Methods

### 2.1. ZIKV Biological Specimens and Virus Isolates

Blood, urine, saliva, and breast milk samples were obtained in February 2016 from a patient infected with ZIKV, two days after the onset of symptoms. The patient presented mild symptoms, maculopapular rash, pruritus, conjunctivitis, arthralgia, malaise, mild fever, and headache. ZIKV diagnosis was performed at the Acute Febrile Diseases Clinic at the Oswaldo Cruz Foundation. The institutional review boards at Fundação Oswaldo Cruz (Fiocruz) approved the study protocol (88551218.6.0000.5262). Breast milk was collected into a sterile container after asepsis of the areola and nipple. The specimens were immediately transported to the Flavivirus Molecular Biology Laboratory. The breast milk fluids were diluted 1:2 in culture medium, filtered in a 0.22 µM pore, aliquoted, and frozen for further analysis.

### 2.2. Virus Isolation from Breast Milk

The African green monkey kidney (Vero) cell line obtained from ATCC—CCL81 was grown at 37 °C, under an atmosphere containing 5% CO_2_, in a culture medium consisting of Earle’s 199 medium (Thermo Fisher Scientific, Waltham, MA, USA) supplemented with 5% fetal bovine serum (FBS; Cutilab), 40 μg/mL of gentamicin (Thermo Fisher Scientific) and 0.5% sodium bicarbonate (Sigma- St. Louis, MO, USA). The Vero cells were seeded at a density of 40,000 cells/cm² in 25 cm² culture flasks 24 h before the inoculation. The diluted (1:2) breast milk sample was thawed on ice and 1 mL of this dilution was inoculated onto a Vero cell monolayer. After 10 min of incubation, the inoculum was replaced by 10 mL of culture medium as a negative control for this experiment, 1 mL of culture medium was used instead of the inoculum in Vero cells seeded in another culture flask. The presence of infectious viral particles was controlled by observation of cytopathic effects (CPE) using an optical microscope. ZIKV Rio-BM1 isolate genome was previously elucidated and is available in the Genbank database (accession number: KY272991). ZIKV isolates Rio-U1 (GenBank: KU926309.2) and Rio-S1 (GenBank: KU926310.2) were previously identified, sequenced, and isolated from urine and saliva, respectively [13].

### 2.3. Cell Lines and ZIKV Infection

Initially, we established the cell permissiveness to ZIKV infection testing different cell lines listed in Table S1. IMR-32, SK-N-AS, THF, THPN, and ST-88-14 cells were maintained in Modified Eagle’s Minimum Essential Medium (DMEM, Thermo Fisher Scientific- Waltham, MA, USA) supplemented with 10% FBS and penicillin (100 IU/mL)—streptomycin (100 µg/mL). Arpe-19 cells were maintained in Modified Eagle’s Minimum Essential Medium with Nutrient Mixture F-12 (DMEM/F-12, Thermo Fisher Scientific- Waltham, MA, USA) supplemented with 10% FBS and penicillin (100 IU/mL)—streptomycin (100 µg/mL). LL-5 and Aag2 cells were maintained in Schneider’s Drosophila Medium. Mammalian cells were incubated at 37 °C, and 5% CO_2_ and insect cells were incubated at 28 °C. Every 3–4 days, the cells were split 1:4 and passed to new culture flasks or seeded into flasks, plates, or slides used in the experiments (described in each section).

For infection, cells were seeded in culture flasks at pre-determined cell density and washed once with a culture medium. The ZIKV stock was diluted to the desired multiplicity of infection (MOI) and added to the cells. The cells were incubated with the virus for 1 h at the appropriate temperature with gentle agitation every 15 min. The inoculum was then replaced with a culture medium and the cells were incubated at the appropriate conditions for the duration of the experiment. As a control, cells were incubated with the culture medium, referred to in the present study as mock-infected cells.

### 2.4. Primary Cultures of Chicken Embryo Fibroblasts (CEF)

Primary cultures of chicken embryo fibroblasts (CEF) were used for the cultivation of Rio-BM1, Rio-U1, and Rio-S1 viruses. For the growth and maintenance of CEF cells in the laboratory, we employed the protocols described in Hernandez et al., 2010 [27]. Briefly, cells were maintained in Minimum Essential Eagle medium (MEM- Gibco, Waltham, MA, USA) supplemented with 10% FBS and penicillin (100 IU/mL)—streptomycin (100 µg/mL).

### 2.5. Virological Characterization

Initially, an infection kinetics assay was performed at the MOI of 0.02 for 24, 48, 72 and 96 h post-infection (h.p.i) to select the best time point to carry out the biological characterization experiments in the different cell lines. The cells were seeded in a culture flask, and after the period of incubation, the cell supernatants were collected for virus titration by plaque-forming assay, and the total RNA was extracted from cells for subsequent ZIKV genome RNA quantification. For all the experiments, the mock-infected cells were used as the negative control for the infection. The statistical analyses were performed using one-way ANOVA followed by Bonferroni’s multiple comparisons test. The differences were considered significant at *p* ≤ 0.05.

### 2.6. Virus Quantification

The viral load from the culture supernatants of ZIKV-infected cells was determined by plaque-forming assays in Vero cells. Briefly, six 10-fold serial dilutions of the virus were inoculated on m the inoculum was replaced by monolayers of Vero cells at 37 °C for 1 h. After that, 1 mL of carboxy-methylcellulose (CMC) at 2.4% diluted in supplemented Earle’s 199 medium. The cells were maintained at 37 °C for seven days. For plaque counting, the cells were incubated overnight with 10% formaldehyde (Merck- Darmstad, HE, GE) washed, and stained with 0.4% crystal violet (Sigma, St. Louis, MO, USA). This experiment was repeated three times.

### 2.7. Immunolabeling

Seventy-two hours following infection of Vero cells on microscope slides, ZIKV-infected, and mock-infected cells were fixed with 4% paraformaldehyde in PBS for 10 min at room temperature. Slides were blocked and permeabilized by incubation with 1% BSA and 0.5% Triton X-100 for 15 min and then incubated for 1 h at room temperature with the monoclonal antibody (Mab) 4G2 (Biomanguinhos—FIOCRUZ, Rio de Janeiro, RJ, BR), which is specific to the flavivirus envelope protein. Cells were washed with PBS and incubated for 60 min at room temperature with secondary antibody Alexa 546 goat anti-rabbit (Thermo Fisher Scientific). Slow fade antifade DAPI (Molecular Probes) was used to stain the nucleus. Preparations were examined with an Olympus IX51 microscope.

### 2.8. RNA Isolation and RT-PCR

Viral RNA was isolated from 140 μL of each biological specimen and cell culture supernatant using the QIAamp Viral RNA Mini Kit (Qiagen- Hilden, NW, GE) according to the manufacturer’s recommendations. RNA was stored at −80 °C until use. The concentration and purity of each RNA sample were measured by Thermo Scientific NanoDrop 8000 Spectrophotometer and Agilent 2100 Bioanalyzer using the Agilent RNA 6000 Nano Kit following manufacturer’s instructions.

The viral RNA was reverse transcribed using the Superscript IV First-Strand Synthesis System (Invitrogen) and random hexamers according to the manufacturer’s recommendations. The reverse transcription reaction was carried out at 23 °C for 10 min, 55 °C for 10 min, and 80 °C for 10 min. Further, the obtained cDNA was amplified by conventional PCR to perform the detection of ZIKV, Chikungunya virus (CHIKV), and Dengue virus (DENV) using GoTaq Green Master Mix (Promega- Madison, WI, USA). The set of primers utilized in this procedure was: ZK3F, 5′ GCTACTGGATTGAGAGTGAGAAG 3′, and ZK2R, 5′CTCAGAGATGGTCCTCTTGTTC3′ for ZIKV; CHIK_E1F, 5′TACCCATTCATGTGGGGC3′ and CHIK_E1R, 5′GCCTTTGTACACCACGATT 3′; DENV, 5′TCAATATGCTGAAACGCGCGAGAAACCG3′ and DENR, 5′ TTGCACCAACAGTCAATGTCTTCAGGTTC3′ for DENV and YFV F 5′-CTGTGTGCTAATTGAGGTGCATTG-3 and YFV R 5′-ATGTCATCAGGCTCTTCTCT-3′ for YFV [28,29,30]. The thermocycling program set up in a Veriti 96 Well thermocycler (Applied Biosystems) was 1 cycle of 95 °C for 5 min; 40 cycles of 95 °C for 40 s, 50 °C for 40 s, 72 °C for 30 s; 1 cycle of 72 °C for 10 min and hold of 4 °C. Ten µL of amplified products were detected by electrophoresis on a 2% agarose gel, visualized by ethidium bromide staining using a UV light.

### 2.9. Plaque Phenotype Assay

One day prior to infection, Vero cells were seeded in six-well plates at a density of 50,000 cells/cm^2^ and incubated at 37 °C with 5% CO_2_. For infection, the medium was replaced with 200 µL of 10-fold serial dilutions of viruses with periodic gentle agitation to facilitate virus adsorption. After 1 h of incubation, viruses were removed and replaced with 3 mL of fresh supplemented Earle’s 199 medium containing 0.5% agarose (Invitrogen). After five days of incubation, cells were fixed with 10% formaldehyde, washed, and stained with 0.4% violet crystal to visualize plaques. Images of the plates were acquired and analyzed using ImageJ software to measure plaque areas. The results were plotted into graphs and statistically analyzed using GraphPad Prism software 8. Statistical tests employed were Kruskal-Wallis with Dunn’s multiple comparison test.

### 2.10. Cell Viability Assay

Vero cells were seeded in 96-well plates at a density of 20,000 cells/well one day before infection. The viral inoculum was prepared in supplemented Earle’s 199 medium. Cell supernatant was removed and replaced by 100 µL of viral inoculum at MOI 0.02, followed by 1 h incubation at 37 °C, 5% CO_2_. After that, the inoculum was removed, and 90 µL of supplemented Earle’s 199 medium was added to each well. After 24 h, 10 µL of Presto Blue Reagent (Invitrogen) was added to each well and incubated for 15 min at 37 °C. Absorbance measures were acquired with SoftMax Pro 6.5 software using VersaMax Tunable Microplate Reader (Molecular Devices- San José, CA, USA), at wavelength 570 nm normalized at 600 nm. Data were analyzed in GraphPad Prism software 8 with One-Way ANOVA followed by Tukey’s multiple comparisons test.

### 2.11. Viral Infection in Cells Treated with Type I Interferon

Vero cells were seeded in 24-well plates at a density of 50,000 cells/cm² one day prior to infection. The next day, cells were treated with IFN alpha (α; PBL Assay Science- Piscataway, NJ, USA) or beta (β; R&D Systems- Minneapolis, MN, USA) at concentrations of 10 UI/mL, 50 UI/mL, 100 UI/mL, or 1000 UI/mL for 6 h before infection. Cell supernatant was removed, and 100 µL of viral suspensions were added at MOI of 0.1 for 1 h of incubation with gentle agitation every 15 min. After that, the virus inoculum was removed and replaced with 0.3 mL of supplemented Earle’s 199 medium containing different IFN concentrations and incubated at 37 °C and 5% CO_2_. Twenty-four hours later, the supernatants were collected for viral titration by plaque assay. The log_10_ of viral titers under treatment with IFNα or IFNβ were normalized with the values obtained from non-treated infected cells. Data obtained from three independent assays were analyzed in GraphPad Prism 8 software. IC_50_ values were calculated from the nonlinear regression function provided by the software (Inhibitor vs. normalized response—Variable slope). IC_50_ values were analyzed by One-Way ANOVA with Bonferroni’s multiple comparison test.

### 2.12. Mouse Model Infection

AG129 mice (deficient in IFN-α/β and IFN-γ receptors) were obtained from the Institute of Science and Technology in Biomodels (ICTB, Fiocruz- Rio de Janeiro, RJ, BR). The animals were bred and maintained under specific pathogen-free conditions. The study was carried out in strict accordance with the Guide of the National Council for Control of Animal Experimentation (CONCEA). The protocol was approved by the Committee on the Ethics of Animal Experiments (CEUA) of the Oswaldo Cruz Foundation (Permit Number: L-034/19). For morbidity/mortality studies, nine to ten-week-old mixed-sex mice were infected by the isolates Rio-U1, Rio-BM1, and Rio-S1 in both hind footpads with 1 × 10^4^ plaque-forming units (PFU) in 60 µL (30 µL/footpad). Mock-infected mice received diluent medium (Earle’s 199 medium supplemented with 25 mM HEPES). Following infection, mice were monitored twice daily for 16 days with the assessment of clinical signs of disease and weight measurement. Submandibular blood withdrawals were performed every two days to monitor viremia. Mice were euthanized when severe clinical evidence of disease was observed according to the signs of morbidity described in Table S2. The evaluated symptoms included difficulty in movement, hunched stance, ruffled fur, aggressiveness, tremors, dyspnea or tachypnea, and weight loss. The final blood collection was obtained by cardiac puncture while the mouse was under deep anesthesia. The animal was immediately euthanized after blood collection by cervical dislocation, and the brain was surgically collected. For viral load determination, brain specimens and whole blood were collected in RNA later (Thermo Fisher Scientific, Waltham, MA, USA) and further extracted with RNAqueous-4PCR Total RNA Isolation Kit (Thermo Fisher Scientific, Waltham, MA, USA) and QIAamp Viral RNA Mini Kit (Qiagen, Hilden, NW, GE), respectively, according to the manufacturer’s instructions. Real-time RT-qPCR was performed using TaqMan Fast Virus 1-Step Master Mix (Applied Biosystems, Waltham, MA, USA) in an Applied Biosystems StepOnePlus Instrument as previously described [13]. Average survival time (AST), percentage of mortality, clinical scores, and body weight loss were calculated and analyzed in GraphPad Prism 8 software. Statistical analysis of Kaplan-Meier survival curves was performed by log-rank test (Mantel-Cox).

### 2.13. Amplicon Based Illumina-Sequencing

The amplicon-based approach involves amplifying a target genome fragment using specific primers before library preparation and sequencing. This study performed high-throughput sequencing using an amplicon-based method of ZIKV Rio BM1, Rio-S1, and Rio-U1 isolates. Prior to Illumina sequencing, cDNA was synthesized by SuperScript IV Reverse Transcriptase kit (Thermo Fisher, Waltham, MA, USA), using random hexamers (Promega, Madison, WI, USA). Virus-specific PCR was performed using a Platinum Taq DNA Polymerase Kit (Thermo Fisher, Waltham, MA, USA). Consensus sequences were also obtained using the conventional Sanger sequencing method [31]. Primers used to amplify eight overlapping PCR ZIKV fragments were previously described [32]. The DNA library preparation was performed using the Nextera XT DNA Library Prep Kit (Illumina, San Diego, CA, USA), according to the manufacturer’s protocol. High-throughput sequencing was performed with MiSeq Reagent Kit v3 (300-cycle, Illumina) to generate 10–25 million 150-nucleotide paired-end reads per sequencing run, using the proprietary Illumina MiSeq System. Raw data from Illumina sequencing were submitted to the Sequence Read Archive (SRA), BioProject ID: PRJNA760935.

### 2.14. Assembly, Variation Calling and Annotation

Illumina paired-end reads were processed using Trimmomatic v. 0.39 [33]. To remove adapter sequences and trim low-quality base pairs. A universal set of Illumina adapters was used as a reference for the adapter removal. Values used for each selected Trimmomatic parameter were chosen based on raw Fastq files after visualizing them with the FastQC program [34]. Briefly, we set the maximum mismatch count to 2, palindrome clip threshold to 30 and simple clip threshold to 10, leading and trailing were set to 3, and applied a sliding window trimming of 4:15, cutting the read if the quality score of 4 contiguous bases made the average score drop below 15. In addition, the MINLEN parameter was set to 36, which means that reads below this specific length are eliminated for the final read set. The final set of reads obtained after trimming was aligned to an appropriate reference genome using the Burrows–Wheeler Alignment tool (BWA) v. 0.7.17–r1188 software [35]. Genome sequences retrieved from GenBank under accession numbers KY272991, KU926310, and KU926309.2 were used to map reads obtained from Rio-BM1, Rio-S1, and Rio-U1 isolates, respectively. SAMtools v. 1.12 was used to convert the output of BWA from SAM to BAM format, as well as to sort and generate indices for the BAM files [36]. Generated BAM files were used for variant calling using LoFreq software version 2.1.5 [37], and single nucleotide variants (SVNs) were annotated using snpEff software version 5.0e [37]. LoFreq is a fast and sensitive variant-caller for inferring SNVs from next-generation sequencing data that returns a VCF file as output. SnpEff uses the LoFreq output and performs SVNs annotation to determine the impact of a detected variant on the sequence; this step adds a new column on the VCF file with the predicted effect on the genome. This software requires a database where the reference sequences must be; as ZIKV reference sequences were not in the default database downloaded from the software page, we built a new database adding ZIKV reference strains: KY272991 (RIO-BM1), KU926310 (Rio-S1), and KU926309.2 (Rio-U1), following the user’s manual (https://pcingola.github.io/SnpEff/se_buildingdb/, accessed on 13 December 2021).

### 2.15. Phylogenetic Analysis

Brazilian ZIKV complete genome sequences were retrieved from the GenBank database and aligned together with other strains using Ali View v.1.27 [38]. Phylogenetic reconstruction was conducted using IQ-TREE software multicore version 2.1.2 [39]; it inferred the Maximum Likelihood tree from the input alignment with the best fit model automatically selected by Model Finder [40]. The ML analysis was carried out with 1000 bootstrap replicates, and the tree was edited using FigTree v.1.4.4 (http://tree.bio.ed.ac.uk/software/figtree/, accessed on 13 December 2021).

## 3. Results

### 3.1. ZIKV Rio-BM1 Isolation

The ZIKV isolates reported in this study were obtained from the urine (ZIKV Rio-U1), breast milk (ZIKV Rio-BM1), and saliva (ZIKV Rio-S1) of three Brazilian patients from the Rio de Janeiro Metropolitan Region during the 2016 epidemic. The isolation and genetic characterization of ZIKV Rio-U1 and ZIKV Rio-S1 were previously reported elsewhere [13].

ZIKV-BM1 was isolated from the breast milk of a puerperal mother on the second day of clinical symptom appearance. The patient was also positive for ZIKV RNA in urine and saliva but not in blood (Figure S1). The ZIKV-positive fluids were negative for Chikungunya (CHIKV), Dengue (DENV), and Yellow Fever (YFV) viruses (Figure S1). Notably, the puerperal mother’s highest viral load was found in urine (6860 ZIKV RNA copies/mL). Saliva and breast milk presented lower viral loads of 217 and 697 ZIKV RNA copies/mL, respectively.

**Figure 1:**
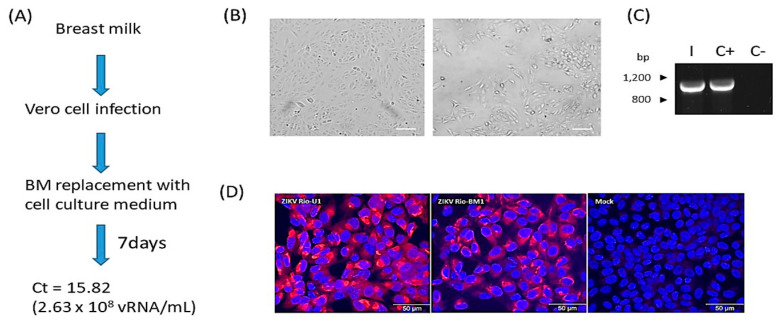
Isolation of ZIKV from the breast milk in Vero cells. (**A**) Experimental design of the isolation of ZIKV from breast milk showing (**B**) the occurrence of CPE after seven days post-infection. Scale bar, 25 µm (**C**) Viral RNA detection in the cell supernatant using RT-PCR/RT-qPCR at seven days post-infection. (**D**) Recognition of ZIKV antigens in Vero cells infected with the ZIKV isolate Rio-BM1. After 72 h of infection, Vero cells were immunostained with the pan-flavivirus monoclonal antibody 4 G2 (red) and counterstained with DAPI (blue). As control conditions, cells were infected with ZIKV isolate Rio-U1 and mock-infected.

Initially, we intended to establish whether infectious viral particles were present in the breast milk of the ZIKV-infected mother. For this, we used a viral adsorption step on Vero cells of a maximum of 10 min to limit the cell exposure to the breast milk sample in order to prevent cell lysis (Figure 1A). Using this method, it was possible to visualize cytopathic effect (CPE) after seven days post-infection, indicating viral proliferation, which was confirmed by detecting a ZIKV-specific 941 bp amplicon by RT-PCR from the cell supernatant (Figure 1B,C). The viral load corresponded to C_t_ = 15.82. Further investigation showed a similar perinuclear labeling pattern with pan-flavivirus monoclonal antibody 4 G2 compared to ZIKV Rio-U1 (Figure 1D). Despite several efforts, it was not possible to isolate ZIKV from the urine and saliva specimens of the patient.

### 3.2. Genomic Analysis

After two rounds of Vero cell passages, the three ZIKV isolates were fully sequenced by next-generation sequencing using the Illumina Miseq platform. All ZIKV full-length genome sequencing resulted in a 10,807 nucleotide (nt) assembled sequence; this sequence contained an ORF of 10,269 nucleotides in length with 5′ (107 nt) and 3′ (431 nt) untranslated regions (UTRs). The comparison of the consensus genome of each isolate revealed a high similarity in nucleotide and amino acid sequences. Nevertheless, some mutations lead to amino acid variations among the isolates (Table 1).

ZIKV isolates shared 99.7–99.9% amino acid identity. Comparing the three ZIKV isolates consensus sequences revealed eight amino acid polymorphisms occurring in the ZIKV precursor polyprotein. One occurred in E protein and the other seven variations consisting of conservative amino acid changes, localized in non-structural proteins, specifically in the NS1, NS2A, NS2B, NS3, NS4A, and NS5 coding regions (Table 1). We aligned the three ZIKV genomes with 605 complete ZIKV genome sequences available at GenBank (sequences of isolates with complete CDS, collected in May 2021). We selected 387 American, 185 Asian, and 33 African ZIKV complete coding sequences.

The E protein amino acid alteration observed in the ZIKV Rio-S1 (polyprotein position 625; E T335A), displays a change from a polar (Threonine) to a non-polar (Alanine) residue. The residue Threonine at position 335 is highly conserved and is located on the surface of the E protein domain III in the infective viral particle. At position E335, only three other genomes displayed amino acid change to Arginine, a negatively charged residue (Table 1). ZIKV Rio-S1 coding sequence exhibits a higher number of non-structural proteins variants than the other two ZIKV isolates. The NS1 349 M→V variation is observed in 28% of American strains and the Methionine residue in all African and Asian sequences and the rest of American genomes (except two with Threonine in this position). In NS3 537 R→K, Lysine is the almost ubiquitous residue in this position, while Arginine occurs only in ZIKV Rio-U1 and Rio-S1 genomes. NS4A 3 A→T polymorphism observed in Rio-S1 exhibited a low frequency and was detected in three American ZIKV genomes from Puerto Rico (N = 2) and Colombia (N = 1). The variation observed in NS5 168 V→A, arises in a highly conserved position, being Alanine present only in the ZIKV Rio-S1 genome.

The single amino acid difference observed in ZIKV Rio-U1 is the variation in NS2B 32 M→I. This polymorphism is also detected in seven precursor polyproteins (in four American strains and three Asian genotypes) (Table 1). The ZIKV Rio-BM1 isolate contains two unique amino acid alterations that are not present in the other 607 ZIKV genomes. To verify if these changes were present in the wild type of virus originally present in the obtained biological sample, the regions containing the observed polymorphism were amplified and sequenced, revealing that the change in NS1 belongs to the wild type virus but not the NS2A alteration.

**Figure 2:**
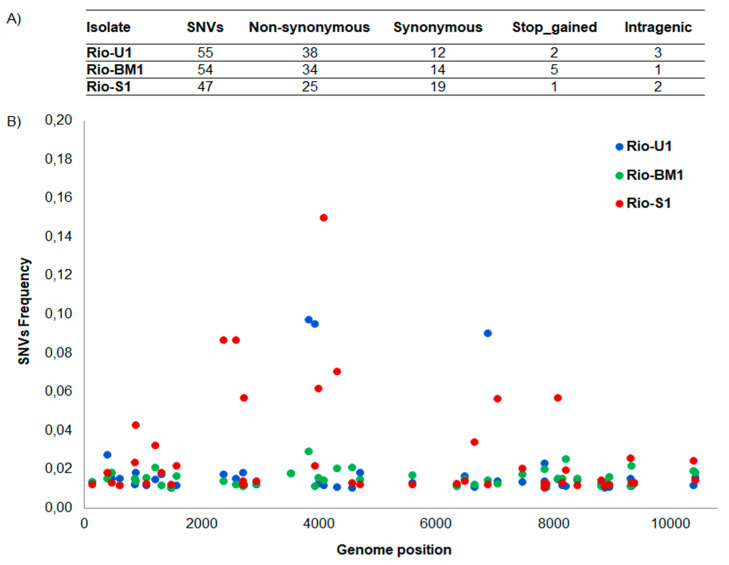
Characterization of single nucleotide variants (SNVs) in the three ZIKV isolates with frequency value >0.01. (**A**) Number of SNV types observed. (**B**) Genome position of SNVs spanning the genome of the three isolates.

To better analyze the genetic variability of the three ZIKV isolates NGS analysis was performed. We established coverage plots using weeSAM v.1.5 (http://bioinformatics.cvr.ac.uk/blog/weesam-version-1-5/, accessed on 13 December 2021). This tool was used to obtain the multiple genome coverage statistics (data not shown). ZIKV isolates genome coverage ranged from 99.8 to 100%, with an average depth ranging from 6385 to 14,328. Quality passed reads were aligned to the consensus sequence, and single-nucleotide variants (SNVs) with frequency >0.01 were counted (Figure 2). In addition, the three isolates shared 99.6–99.7% of nucleotide identity, showing variations in fifty-five positions (Figure 2). The genetic comparison shows Rio-S1 isolate is the most diverse among the studied isolates regarding the number of SNVs.

Among SNVs with >0.01 frequency value, frequencies ranged from 0.010117 to 0.028632 (Rio-BM1), 0.010065 to 0.096905 (Rio-U1) and 0.010014 to 0.158707 (Rio-S1) (Figure 2). Isolates analyzed in the current study shared eight non-synonymous SNVs (Table 2) (The blue color represents the Rio-U1 virus, the red color Rio-S1 and the green Rio-BM1 virus). In addition, Rio-BM1 and Rio-U1 isolates shared eight other non-synonymous SNVs (genome positions: 1419, 2742, 3345, 4783, 5569, 7411, 7771, 8735, 8873), Rio-U1 and Rio-S1 isolates shared four additional non-synonymous SNVs (genome positions: 2719, 6363, 8419, 8902), and Rio-BM1 and Rio-S1 isolates shared only one more non-synonymous SNV (genome position 7860). The total number of SNVs as well as the positions of synonymous, non-synonymous (missense), and stop-gained variants were plotted on the viral genome for each isolate (Figure 2). Most of the non-synonymous observed SNVs occurred inside non-structural proteins (Figure S3).

### 3.3. Phylogenetics

ZIKV phylogenetic analysis was performed using 46 Brazilian strains isolated between 2015 and 2017. Following previous studies [41,42], all Brazilian strains showed close phylogenetic relationship grouping in a significant cluster (bootstrap score 100%) together with strains from the Asian lineage retrieved from the Genbank, as well as other ZIKV isolates. (Figure S2). Isolates Rio-BM1 and Rio-U1 were grouped in the same branch, whereas isolate Rio-S1 were grouped separately.

### 3.4. Viral Characterization in Cell Culture

**Figure 3:**
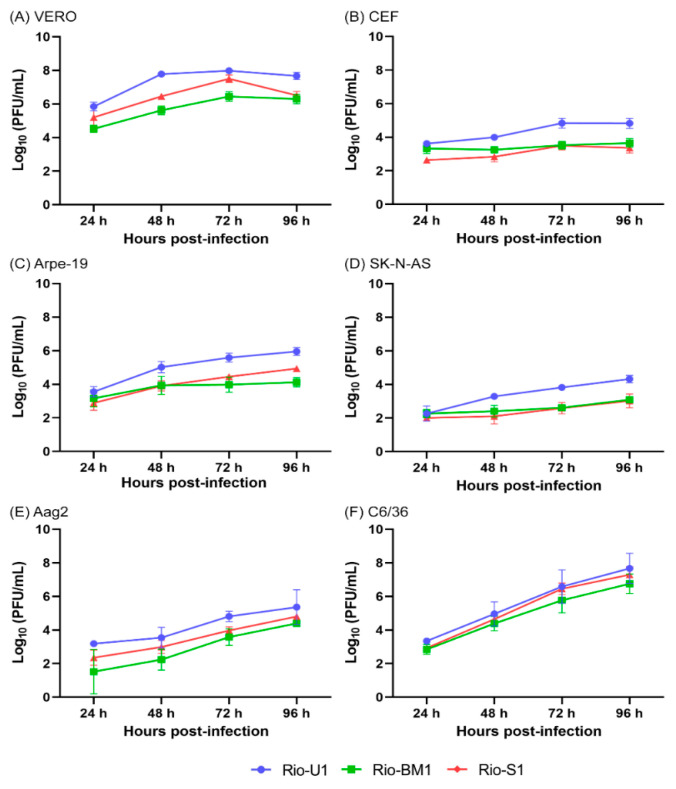
Viral replication of ZIKV Rio-U1, Rio-S1, and Rio-BM1 in different cell lines. The replication profiles were obtained from (**A**) Vero, (**B**) chicken embryo fibroblasts (CEF); (**C**) Arpe-19; (**D**) SK-N-AS; (**E**) C6/36 and (**F**) Aag2 cell lines. Cells were infected with ZIKV isolates at MOI 0.02, and supernatants were used to determine viral titers each day post-infection for 4 days. Statistical analyses applied were One-Way ANOVA with Bonferroni’s multiple comparison test (Figure S4) using GraphPad Prism 8.

To study the biological characteristics of the ZIKV Brazilian isolates Rio-U1, Rio-BM1, and Rio-S1, different mammalian and insect cell lines were used (Table S1). The ability to infect different cell lines was evaluated by measuring the viral titer (PFU/mL) after viral replication in cell cultures (Figure 3). The Rio-U1 isolate produced more viral particles, and Rio-BM1 tends to replicate less in mammalian cells (Vero, Arpe-19, and SK-N-AS). The difference was less evident in insect cell lines, such as Aag2 and C6/36 cells, with highly similar replication profiles in the latter. The viral proliferation peak was reached at 72–96 h post-infection. The titer and standard deviation details achieved at the peak of infection are described in Table S3.

### 3.5. Plaque Morphology

**Figure 4:**
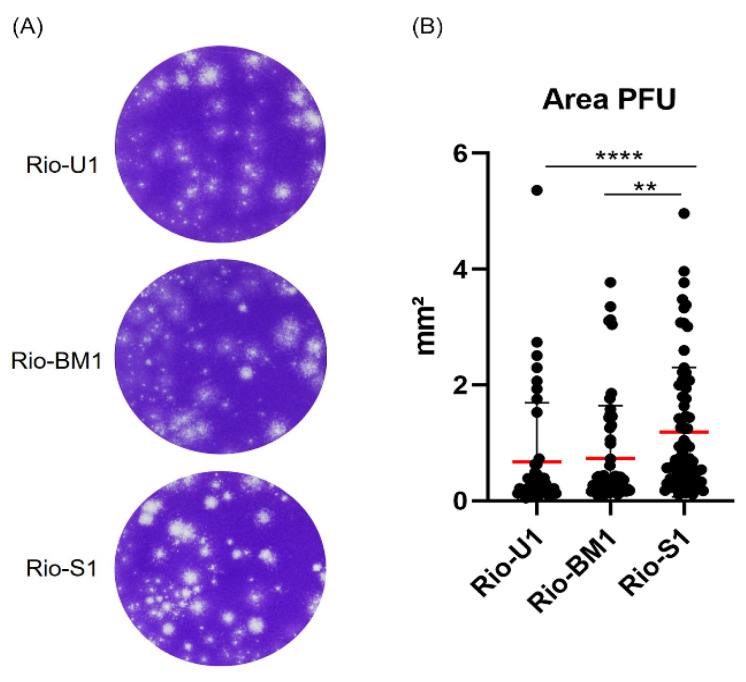
Plaque size phenotype of ZIKV isolates Rio-U1, Rio-BM1 e Rio-S1. (**A**) Infected Vero cell monolayer stained with crystal violet. (**B**) Scatter plot graphs indicating the individual values for plaque area. Red bars represent the median area. ** represents *p* ≤ 0.01, and **** represents *p* ≤ 0.0001.

To characterize the infection pattern of these different isolates, we compared viruses through plaque morphology in Vero cells. As seen in Figure 4, the morphology observed in Rio-U1 and Rio-BM1 exhibit smaller and less diverse plaque sizes (average plaque areas: 0.67 ± 1.01 mm² and 0.73 ± 0.90 mm², respectively), whereas the pattern observed in Rio-S1 is larger and with more morphologically diverse plaque phenotypes (average plaque areas: 1.18 ± 1.11 mm²). The statistical analysis of the plaque area median revealed that Rio-U1 and Rio-BM1 do not show significant differences, however, when compared with Rio-S1, they are significantly smaller (Rio-S1 to Rio-U1 *p* < 0.0001, and Rio-S1 to Rio-BM1 *p* = 0.0032).

### 3.6. Cell Viability Assay

**Figure 5:**
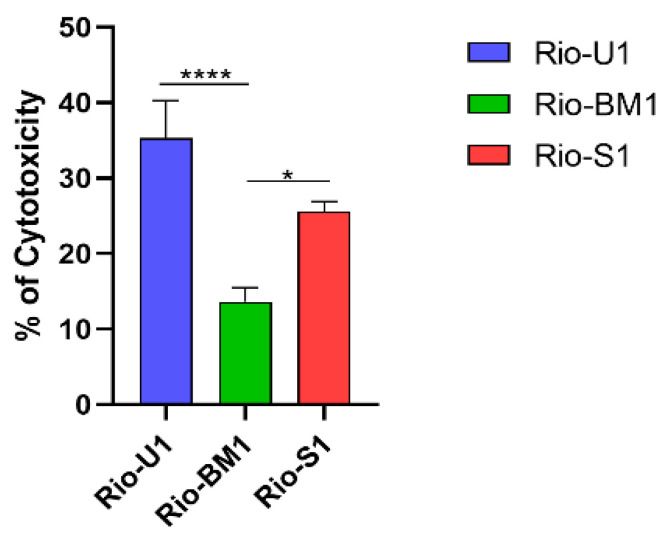
ZIKV-induced cytotoxicity in Vero cells at 24 h post-infection at MOI 0.02. Statistical analyses were performed in GraphPad Prism 8, by Ordinary One-Way ANOVA with Tukey’s multiple comparisons tests: * represents *p* ≤ 0.05, and **** represents *p* ≤ 0.0001.

We next tested ZIKV-mediated cytotoxicity of the three ZIKV isolates in Vero cells, as viruses demonstrated different cell infection capabilities. After 24 h of infection at MOI 0.02, the cell viability was determined (Figure 5). The viruses Rio-U1 and Rio-S1 induced 35.3% and 25.5% cell mortality, while Rio-BM1 induced significantly lower levels of cytotoxicity (approximately 13.6%). These data are in line with the viral replication kinetics in Vero cells, suggesting an association of higher viral proliferation rates with a more significant cell death capacity.

### 3.7. Viral Replication in Vero Cells in the Presence of Type I Interferon

**Figure 6:**
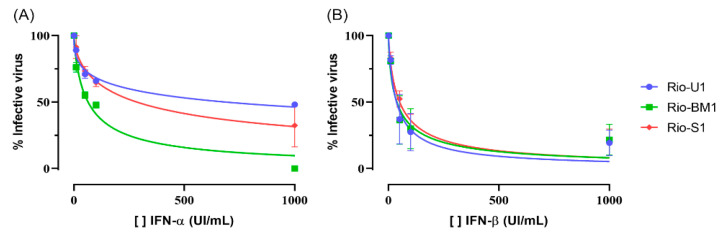
Effect of type I IFN treatment on ZIKV proliferation in Vero cells. The cells were infected at MOI 0.1 for 24 h, and the virus titer in the supernatant was determined by plaque assay. Viral titers under treatment with IFNα and IFNβ were normalized with the values obtained from non-treated infected cells. The graphs represent values of normalized-viral yields obtained for each tested concentration of (**A**) IFN-α and (**B**) IFN-β. Data were analyzed with GraphPad Prism 8 software. The curves were fitted by nonlinear regression of normalized data with variable slope.

The type I IFN system is crucial in combating viral infections in mammalian cells. Therefore, we evaluated the effect of IFN in Vero cells infected by the three isolates. The cells were pre-treated for 6 h with recombinant human IFNα or IFNβ and were infected with an MOI of 0.1. After 24 h, viral concentrations in cell supernatants were determined by plaque titration. Both IFNα and IFNβ strongly and dose-dependently inhibited viral replication, but with different efficacies (Figure 6). The ZIKV isolates appeared to be more sensitive to IFNβ, with IC_50_ of 36.3 IU/mL, 37.5 IU/mL and 50.2 IU/mL for Rio-U1, Rio-BM1, and Rio-S1, respectively (Figure 6). On the other hand, the IFNα treatment revealed more noticeable differences between the viruses. ZIKV Rio-U1 and Rio-S1 had higher viral titers displaying IC_50_ of 684.2 IU/mL and 284.1 IU/mL, while Rio-BM1 had an IC_50_ of 63.7 IU/mL. In the cultures treated with IFNα, the inhibition of viral growth between Rio-U1 and Rio-BM1 are significantly different (*p* = 0.0159). Treatment with IFNβ did not generate a significant difference in the growth profiles of the ZIKV isolates.

### 3.8. Virulence in AG129 Mice

**Figure 7:**
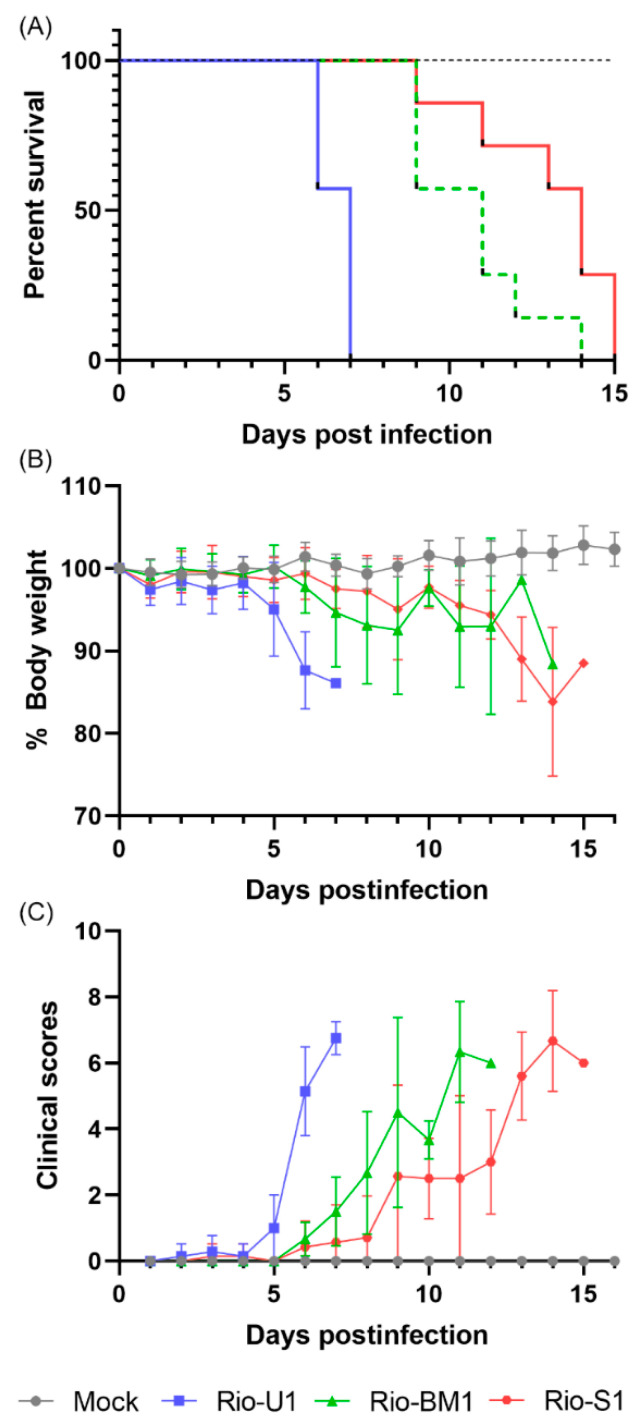
Infection of AG129 mice by the ZIKV isolates Rio-U1, Rio-BM1 e Rio-S1. Mice were infected with 10,000 PFU in both hind footpads with the different viral isolates or with diluent medium (Mock). We followed up with the mice daily for two weeks. (**A**) Deaths were recorded in Kaplan–Meier survival curves, and morbidity among these animals was also determined by (**B**) weight loss and (**C**) clinical scores. Error bars represent the SD of the mean for each group of mice. (The blue color represents the Rio-U1 virus, the green Rio-BM1 and the red Rio-S1 virus).

We compared the infective ability of the three isolates in AG129 mice (deficient in IFN-α/β and IFN-γ receptors). Subcutaneous infection with the isolates (10,000 PFU) led to weight loss and progressive increase in clinical score with disease characterized by movement constraints, hunched stance, aggressiveness, tremors, dyspnea/tachypnea, and weight loss in all three isolates, but varying in time-points. The mouse group infected with Rio-U1 started to lose weight from day five onwards, together with the appearance of clinical signs of disease. The group inoculated with Rio-BM1 displayed the initial clinical signs on day six and the next day started to lose weight. Finally, Rio-S1 also provoked the development of the disease from day six onwards, but with a slower progression, and weight loss was only observed from day nine post-infection (Figure 7). The three isolates triggered the mortality of 100% of the animals but exhibited different average survival times (AST) (Figure 7A). Mice inoculated with Rio-U1 had earlier death (AST of 6.5 ± 0.5 days). Animals who received Rio-BM1 presented AST of 10.7 ± 1.8 days. The longest survival time score was presented in the Rio-S1 group, with an AST of 13 ± 2.2 days. The survival curves were statistically different (*p* < 0.0001) Log-rank (Mantel-Cox) analysis. As expected, uninfected controls did not exhibit weight loss and clinical signs of disease and survived until the experimentation time. These data are in accordance with those derived from cell models, in which Rio-U1 displayed higher viral titers, including funder treatment of IFN-α, and a higher capacity to induce cell death. In conjunction, these results point to a higher virulence of virus Rio-U1 than the other viral isolate counterparts.

**Figure 8:**
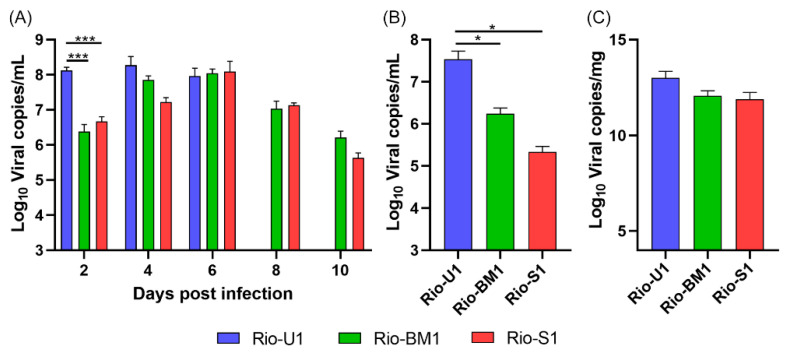
ZIKV viral load after inoculation of AG129 mice with ZIKV isolates. (**A**) Blood viremia in the 2, 4, 6, 8, and 10 days post-infection and at (**B**) the moment of death in the blood and (**C**) in the brain. * Represents *p* ≤ 0.05, and *** Represents *p* ≤ 0.001.

We also determined viremia at days two, four, six, and eight post-inoculations of the ZIKV isolates (Figure 8A) as well as at the time of death (euthanasia) (Figure 8B). Animals infected with Rio-U1 had higher viral loads at all times points analyzed, with the peak viral load of 1.9 × 10^8^ viral copies/mL at day four post-inoculation. Animals infected with the viruses Rio-BM1 and Rio-S1 showed a delayed peak viremia on the sixth day after infection with viral loads of 1.2 × 10^8^ and 1.1 × 10^8^ viral copies/mL, respectively. Statistical analysis revealed a significant difference in viral load between isolates on the second day after infection (*p* < 0.0001. On the eighth day after infection, all animals that received Rio-U1 had already died, hence, it. was only possible to collect blood from the animals in the groups inoculated with the other two viral isolates. At the time of death, we determined differences in viral loads in the blood of animals. The isolate Rio-U1 presented a higher viral load, 3.4 × 10^7^ viral copies/mL, followed by Rio-BM1 and Rio-S1 with 1.7 × 10^6^ and 2.1 × 10^5^ PFU/mL, respectively. Statistical analysis revealed a significant difference between the viral load of ZIKV Rio-U1 and the other isolates (Rio-U1 × Rio-S1, *p* = 0.025, and Rio-U1 × Rio-S1, *p* = 0.020) at the time of death (Figure 8B). Finally, the main target organ of ZIKV infection in the AG129 mouse model is the brain, we measured the viral load in this tissue on the day of death (Figure 8C). The viral loads of animals infected with all ZIKV isolates were exceptionally high, reaching values around 13 log_10_ viral copies/mg with no significant difference between the viruses (*p* > 0.05).

## 4. Discussion

In this study, we performed a comparison of three ZIKV isolates obtained in Brazil during the same epidemic period from breast milk (Rio-BM1), urine (Rio-U1), and saliva (Rio-S1) specimens of three different patients, demonstrating that a few genetic mutations are sufficient to impact the infection performance in in vitro and in vivo biological models.

Since the last major outbreak of ZIKV in the Americas in 2016, three studies reported the detection of infectious viral particles in breast milk samples [14,15,43], demonstrating that ZIKV infection is a concern for the health of breastfeeding mothers and infants Here, ZIKV was isolated from a breastfeeding woman presenting symptoms of ZIKV infection. The virus isolation was only achieved from her breast milk, and even though the patient’s urine sample tested positive for ZIKV and showed a higher viral load, isolation of the virus from this specimen was not successful. Despite the detection of ZIKV RNA and infectious ZIKV particles in breast milk, the mother continued breastfeeding. The newborn of this mother did not show any symptoms of infection and had normal development, which may indicate that the virus might have either caused asymptomatic infection or not infected the infant. Our findings corroborate previous studies; despite finding active viral particles, they do not support the potential for viral transmission through breast milk.

Viral epidemics can often be associated with the accumulation of one or more mutations in the viral genome [44,45]. Indeed, in general, arboviruses and many other RNA viruses exhibit a unique feature compared to DNA viruses, the lack of proof-reading activity of their RNA-dependent RNA polymerases [46]. It is well known that RNA viruses evolve at higher rates than DNA viruses [47]. However, there is a restriction element associated with RNA viruses transmitted by arthropod vectors to mammals, which is the ability to propagate in both insect and mammal hosts [48]; therefore, fixing changes in viral proteins cannot provoke a substantial loss of fitness in either. Nevertheless, in the Americas, one of the main characteristics of the ZIKV lineage is its rapid spread, consistent with a pattern of intense genetic diversification, as the lineage expands into new territories with immunologically naïve populations [49]. Thus, viral isolates from different patients during the same epidemic period can present unique amino acid variants. Several studies have experimentally investigated the role of some of these amino acid substitutions, and a correlation between genetic changes and the increased pathogenicity of ZIKV in recent outbreaks was suggested [50,51].

The three ZIKV isolates analyzed in the present study belonged to the American cluster inside the Asian lineage. In addition, a comparison of ZIKV isolates with available sequences in the Genbank database revealed that they carry the prM N139, NS1 V982, and NS5 V2634 (numbering from the open reading frame of the ZIKV genome) signature characteristic of the American isolates. The substitutions S139N at prM and A982V at NS1 arose during ZIKV spread from Asia to the Americas and were associated with severe microcephalic phenotype in a neonatal mouse model and increased virus transmission in mosquitoes, respectively [52,53,54]. The M/T2634V substitution at NS5 is believed to be more recent and may impact neurotropism since the relative rate of congenital Zika virus syndrome increased significantly in Brazil compared with French Polynesia [54].

Apart from these putative genetic determinants of virulence, when compared to each other, the three isolates presented eight amino acid differences, seven in non-structural proteins and one inside E protein, in accordance with studies that indicate non-structural proteins as the main targets of positive selection in the flavivirus evolution [55]. It is well known that RNA viruses exist as a cloud of closely related sequence variants, also named quasispecies [56]. To further investigate the genetic variability of ZIKV isolates analyzed in the current study, we identified SNVs using next-generation sequencing. Isolates contained twelve SNVs (frequency value >0.01), being eight non-synonymous mutations occurring inside non-structural proteins, suggesting that they might represent specific adaptive mutations. Of notice, two SNVs showed frequency value >10%, both observed in Rio-S1 isolate, T351A (NS1), and K186R (NS2A). Residue 351 inside NS1 has been previously described as part of a putative epitope [57]. However, to assess the role of each mutation in these isolates, viral clones with point mutations can be constructed, and the aspects already studied can be evaluated.

The isolate Rio-BM1, compared to the two other isolates, displayed the lowest rates of replication in mammal cells, induced lower rates of cell death, and was more susceptible to type I IFN treatment. In the murine model, the isolates Rio-BM1 and Rio-S1 seem to be more attenuated than ZIKV Rio-U1. Rio-BM1 carries two unique amino acid changes compared to the other viruses at NS2A N1277S and NS1 N889S. Furthermore, Rio-BM1 has a single nucleotide substitution inside the 3′UTR region. An intramolecular pairing between the 5′ and 3′UTRs regions facilitates the transformation between genomic RNA conformations conferring a vital role in the coordination of virus replication [58]. Alteration at the 3′UTR could impair the evasion mechanisms or destabilize interactions between the viral non-coding regions. The potential contribution of these amino acid markers to the less virulent phenotype should be further investigated. The variation in NS2A was not present in the wild type of virus obtained from patient samples from urine, saliva, and breast milk, but only in the viral stock after passage in Vero cell cultures. Interestingly, this position is highly conserved in ZIKV, and this alteration was only detected in the Rio-BM1 viral stock. There are some records of adaptative mutations in flavivirus-infected Vero cells [59,60], and it should be the case of NS2A mutation in Rio-BM1.

On the other hand, the Rio-U1 isolate displayed the highest rates of replication in mammal cells, of type I-IFN resistance, and cytotoxicity induction. The pathogenicity of ZIKV Rio-U1 was previously studied in AG129 mice and it was demonstrated that Rio-U1 isolate caused lethality at lower doses than other common ZIKV isolates and provoked a high rate of fetal development complications in rhesus macaques (*Macaca mulatta*) [61]. The only specific Rio-U1 variation occurs in NS2B M1404I. NS2B is a cofactor for NS3 proteinase activity and is critical for NS3-mediated cleavages [62]. Functionality studies of flavivirus NS2B revealed that mutations in the hydrophobic regions had subtle effects on proteolytic processing, while mutations in this conserved protein domain dramatically reduced cleavage efficiency or eliminated all cleavages [63].

The isolates studied here have a greater sensitivity to IFNβ than IFNα. The most effective action of IFNβ has already been described in Huh-7 cell infection by ZIKV. This study hypothesized that the higher susceptibility of ZIKV to IFNβ was due to a more robust induction of ISGs by IFNβ in Huh7 cells compared to IFNα [64]. The literature reports that non-structural proteins present several sites that mediate interaction with the host immune system [65] athwart preventing ISG production by interfering with PRR-mediated IFN production or by directly targeting signaling intermediaries downstream of the IFNAR1/2 receptor [66]. Furthermore, non-structural proteins are also essential for viral polypeptide processing, replication, and modulation of the host [67]. The variations found in ZIKV isolates are localized mainly in non-structural proteins, except for a variation in the envelope protein that is unique to the saliva isolate (Rio-S1); they may modify the viruses in question in different ways.

We investigated the infectivity and virulence of the three isolates in the AG129 mouse model that lack IFN-α/β and IFN-γ receptors due to the viral sensitivity to IFNα and β. Several immunocompromised adult mouse models have been reported to support the replication of ZIKV; among those, AG129 showed greater susceptibility and more severe disease upon ZIKV infection [68,69]. AG129 is a powerful model in flavivirus studies such as DENV, as it replicates the vascular leak-like syndrome with features reminiscent of severe dengue disease in humans and displays DENV tropism similar to humans [70]. Here, we report that all ZIKV isolates can cause morbidity and mortality in AG129 mice, presenting clinical signs characterized by weight loss, difficulty in walking or paralysis, goosebumps, and curved spine, similar to symptoms described in other articles [68,71,72]. In the present study, we demonstrated that the ZIKV isolates were highly replicative in the AG129 mouse brain, as observed previously [73], leading to rapid disease progression. In accordance with the data obtained in vitro, we observed a more virulent phenotype of ZIKV Rio-U1 in this mouse model, causing the death of 100% of animals at an earlier time than that observed for the other two viral isolates.

In conclusion, here, we comparatively studied three Zika virus isolates related to human cases arisen in 2016 in Rio de Janeiro, Brazil. The human cases from which the three viruses were isolated correspond to a short period time of Zika epidemics (January and February 2016). The genetic variability found in the ZIKV isolates, most of it associated with non-synonymous alteration occurring inside viral non-structural proteins, points to a role of these alterations in virulence modulation. This initial study is a starting point to guide further analyses utilizing reverse genetics to elucidate the role of some amino acid polymorphisms in ZIKV virulence.

## Figures and Tables

**Table 1:** Amino acid variations among the 2016 Brazilian ZIKV isolates.

Position		Rio-U1 *	Rio-BM1	Rio-S1	Site Variation in ZIKV
Polyprotein	Protein	(Frequency) **	(Frequency)	(Frequency)	
625	E _335_	T (607)	T (607)	A (1)	R not A in: America MW122427 (Puerto Rico) Africa KU963574 (Nigeria) e HQ234500 (Nigeria)
889	NS1 _95_	N (607)	S (1)	N (607)	S unique occurrence in Rio-BM1—position highly conserved in all ZIKV sequences
1143	NS1 _349_	M (500)	M (500)	V (108)	M in Africa and Asia ZIKV genomes America ZIKV: M (71%); V (28%) and, T (1%)
1277	NS2A _131_	G (607)	S (1)	G (607)	S unique occurrence in Rio-BM1—position highly conserved in all ZIKV sequences
1404	NS2B _32_	I (8)	M (600)	M (600)	KU729217 (Brazil); MF574585 (Colombia) e MF574587 (Colombia); KX156774 (Panama); MK696551 (China) e MF964216 (China); MW015936 (Thailand)
2039	NS3 _537_	R (2)	R (2)	K (606)	K is presented in all ZIKV genomes, except in Rio-U1 and Rio-BM1 and KX830930 (Brazil)
2122	NS4A _3_	A (604)	A (604)	T (4)	MW122434 (Puerto Rico) e MW122427 (Puerto Rico); MF574563 (Colombia)
2688	NS5 _168_	V (607)	V (607)	A (1)	A unique occurrence in Rio-S1—position highly conserved in all ZIKV sequences

* Amino acid variations colored in blue; ** frequency in 608 ZIKV genomes.

**Table 2:** Non-synonymous SNVs among the 2016 Brazilian ZIKV isolates.

Position	Polyprotein Position	Rio U1	Rio S1	Rio BM 1	Protein	aa Change
1419	438				E	H→N
2719	871				NS1	A→E
2742	879				NS1	Q→K
3345	1080				NS1	H→N
3841	1245				NS2A	W→L
3947	1280				NS2A	L→F
4783	1559				NS3	A→E
5569	1821				NS3	P→Q
5618	1837				NS3	D→E
6363	2086				NS3	H→N
6510	2135				NS4A	H→N
7411	2435				NS4B	V→G
7488	2461				NS4B	W→G
7771	2555				NS5	V→G
7860	2585				NS5	L→V
8419	2771				NS5	V→G
8735	2876				NS5	F→L
8842	2912				NS5	P→Q
8873	2922				NS5	N→K
8902	2932				NS5	A→E
9351	3082				NS5	H→N
9390	3095				NS5	Q→K

## Data Availability

Samples are deposited at BioSample Database. Rio-U1, accession: SAMN21239024 (Urine from patient, one passage in Vero cell), Rio-S1, accession: SAMN21239025 (Saliva from patient, one passage in Vero cell) and Rio-BM1, accession: SAMN21239026 (Breast milk from patient, one passage in Vero cell). Data supporting ngs results are openly available in Sequence Read Archive (SRA), reference number: Bioproject ID PRJNA760935.

## Supplementary Materials

The following are available online at https://www.mdpi.com/article/10.3390/microorganisms10050854/s1, Figure S1: Detection and quantification of ZIKV genomic RNA in body fluids; Figure S2: Maximum Likelihood phylogenetic tree of ZIKV based on open reading frame (ORF) sequences; Figure S3: Number of single nucleotide variants (SNVs) occurring in the ORF per gene region of three ZIKV isolates analyzed in the current study; Figure S4: Viral replication kinetics in different cell lines; Table S1: Animal cells susceptibility to Zika virus infection; Table S2: Clinical scores for monitoring signs of disease in AG129 mice; Table S3: Titer and standard deviation at the peak of infection.
