# Supplementary material for: Phenotypic and Genetic Variability of Isolates of ZIKV-2016 in Brazil
Source: Microorganisms. 2022 Apr 21;10(5):854. doi: 10.3390/microorganisms10050854 (PMC9146765; doi:10.3390/microorganisms10050854)
Supplement: Supplementary file 1 [file microorganisms-10-00854-s001.zip › microorganisms-1532537-supplementary.pdf]

microorganisms

MDPI

Supplementary materials

Table S1. Animal cells susceptibility to Zika virus infection.

Cells	Origin	ZIKV
Vero	African Green Monkey kidney	++++
Arpe-19	Human retinal	++++
C6/36	*Aedes albopictus* larva	++++
AaG2	*Aedes aegypti* embryo	+++
SK-N-AS	Human neuroblastoma	++
ST88-14	Human Schawnn cell	++
THF	Human fetal thymus	++
THPN	Human thymus	++
CEF	Chicken embryo fibroblasts	++
IMR-32	Human neuroblastoma	+
LL-5	Sandyfly embryo	–

Cells were infected with the ZIKV isolate Rio-U1 for 72 h and the supernatant was collected. The positivity rate was determined after viral titration and calculated as follows: -: less than 10^2 PFU/mL; +: 10^3 PFU/ml; ++: 10^4 PFU/ml; +++: 10^5 PFU/ml; ++++: 10^6 PFU/ml or more.

Table S2. Clinical scores for monitoring signs of disease in AG129 mice.

Observation	Score
**Body weight**	
Loss of 5-10%	1
Loss of 10-15%	2
Loss of 16-20%	3
Loss of 20% or more	6
**Fur**	
Slightly ruffled	2
Evidently ruffled	3
**Respiration**	
Accelerated breathing (tachypnea)	1
Difficult breathing (dyspnea)	3
**Clinical complications**	
Tension during handling	1
Tremors, breath noises, aggression, vocalizations	3
**Motility**	
Abnormal walking and posture (slightly hunched, reduced activity)	1
Massively abnormal walking and posture (evidently hunched, moderately reduced activity)	2
Motility only after stimulation, isolation, lethargy	3

No activity for more than 24 hours	6
**Measures according to total score**	
**3**	Animals are carefully observed, with monitoring 2 times a day
**6**	Implementation of humane endpoint, abort experiment (euthanasia)

Table S3. Titer and standard deviation at the peak of infection.

	Mean/Standard deviation		
	**Rio-U1**	**Rio-BM1**	**Rio-S1**
**VERO**	7.98 ±0.11	6.45 ±0.28	7.50 ±0.22
**ARPE-19**	5.59 ±0.27	3.98 ±0.45	4.45 ±0.14
**SK-N-AS**	3.82 ±0.12	2.61 ±0.12	2.59 ±0.33
**CEF**	4.84 ±0.30	3.52 ±0.23	3.50 ±0.25
**AAG2**	5.36 ±1.03	4.40 ±0.18	4.80 ±0.14
**C6/36**	7.67 ±0.90	6.75 ±0.58	7.30 ±0.48

(A)

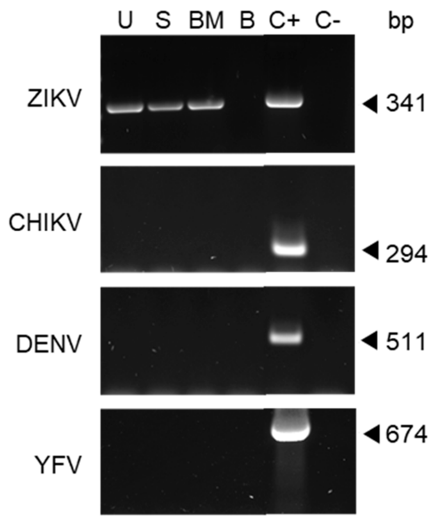

(B)

Specimen	Ct	vRNA copies/mL
Urine	32.13	6.86 x 10^3
Breast milk	35.51	6.97 x 10^2
Saliva	36.50	2.17 x 10^2
Blood	ud	<40

Figure S1. Detection and quantification of ZIKV genomic RNA in body fluids. (A) The profiles obtained from urine (U), saliva (S), breast milk (BM), blood (B) and viral isolate (I) samples. Each set of electrophoretic analysis corresponds to specific primers employed to detect Zika virus (ZIKV), Chikungunya virus (CHIKV), dengue virus (DENV) and Yellow Fever virus (YFV). The lanes (C+) are positive controls in whose the primers were used to amplify the related virus and (C-) is a negative control of amplification. The length of each PCR product is indicated on the right of the figure. (B) Viral load values are expressed as Ct values and as viral RNA copies /ml.

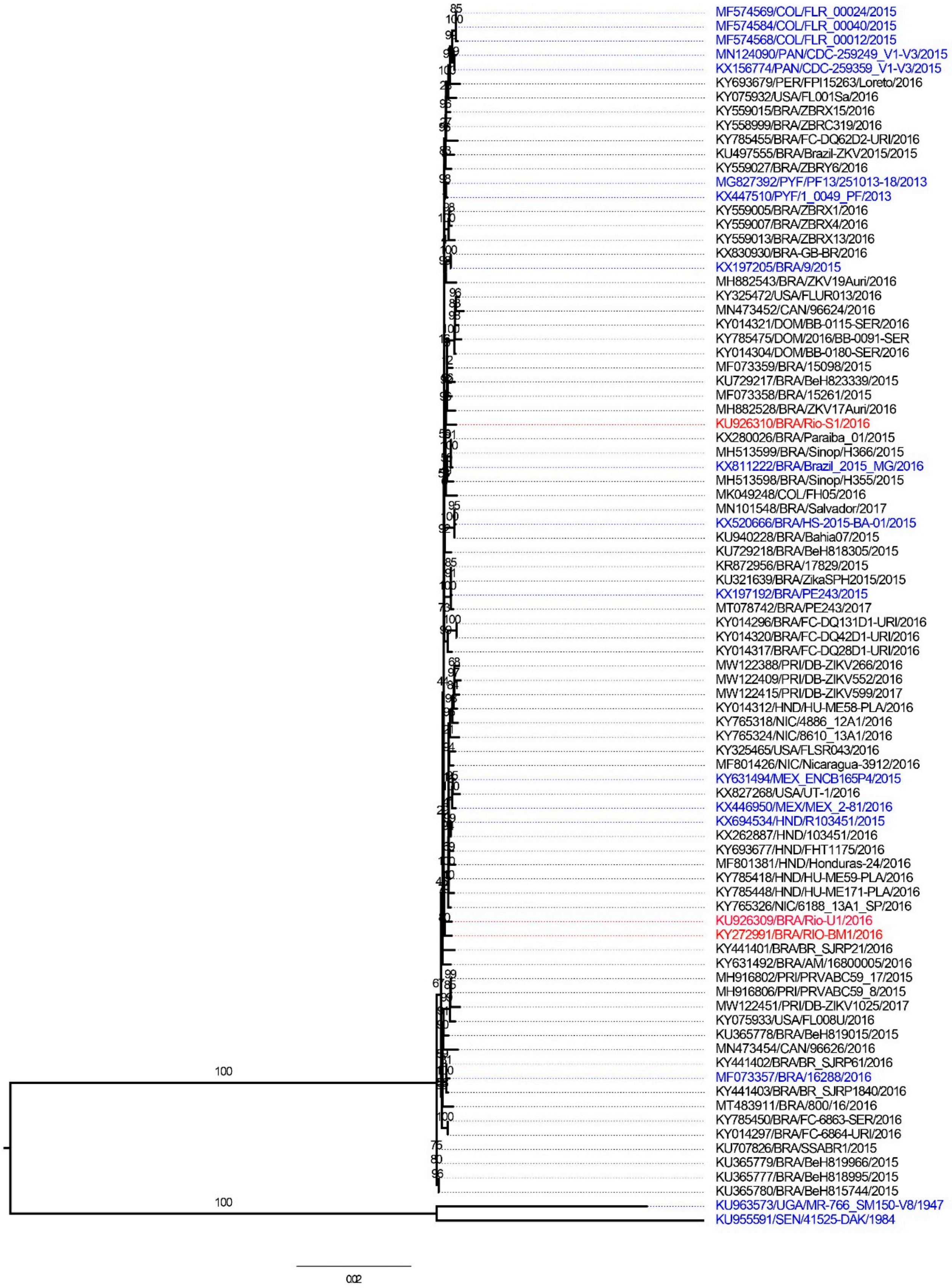

Figure S2. Maximum Likelihood phylogenetic tree of ZIKV based on open reading frame (ORF) sequences. Isolates Rio-BM1, Rio-S1 and Rio-U1 are shown in red. Several ZIKV isolates were used in the phylogenetic tree, identified in blue. Bootstrap values > 70 (1000 replicates) are shown.

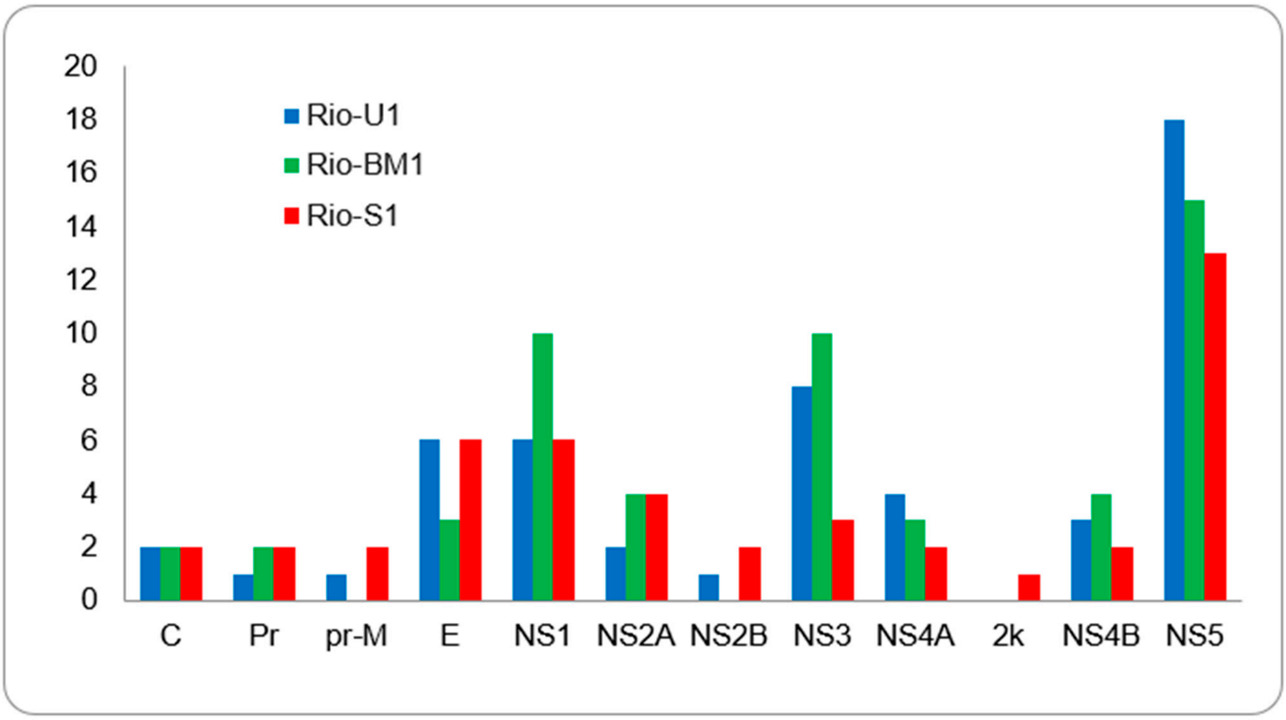

	C	Pr	pr-M	E	NS1	NS2A	NS2B	NS3	NS4A	2k	NS4B	NS5
Rio-U1	2	1	1	6	6	2	1	8	4	0	3	18
Rio-BM1	2	2	0	3	10	4	0	10	3	0	4	15
Rio-S1	2	2	2	6	6	4	2	3	2	1	2	13

Figure S3. Number of single nucleotide variants (SNVs) occurring in the ORF per gene region of three ZIKV isolates analyzed in the current study.

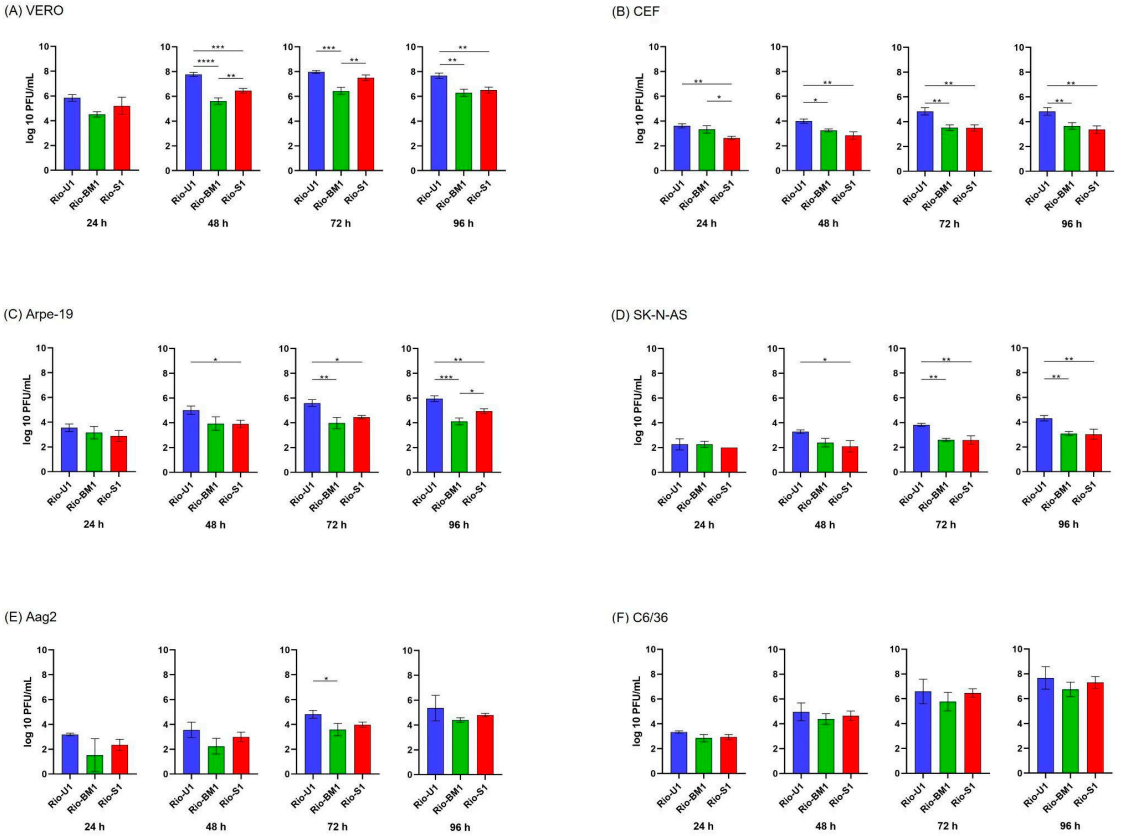

Figure S4. Viral replication kinetics in different cell lines. Statistical analyses applied were One-Way ANOVA with Bonferroni's multiple comparison test. * represents $P \leq 0.05$, ** represents $P \leq 0.01$, *** represents $P \leq 0.001$ and **** represents $P \leq 0.0001$.
